# Health advocacy role performance of nurses in underserved populations: A grounded theory study

**DOI:** 10.1002/nop2.1907

**Published:** 2023-06-14

**Authors:** Luke Laari, Sinegugu Evidence Duma

**Affiliations:** ^1^ School of Nursing and Midwifery, College of Health Sciences University of Ghana Accra Ghana; ^2^ College of Health Sciences University of KwaZulu‐Natal Durban South Africa

**Keywords:** grounded theory, health advocacy, nursing role, nursing theory, underserved populations

## Abstract

**Aim:**

Nurses' health advocacy (HA) role requires them to speak up for patients, clients, and communities in relation to healthcare. Various studies report the importance of the HA role of the nurse in healthcare. However, nurses' performance in this role is not clear yet. The present study aims to identify and explain how nurses perform their HA role in underserved populations.

**Design:**

Qualitative grounded theory by Strauss and Corbin.

**Methods:**

Data were gathered from three regional hospitals in Ghana with 24 registered nurses and midwives as participants through purposive and theoretical sampling techniques. Face‐to‐face in‐depth semi‐structured interviews were conducted from August 2019 to February 2020. The data were analysed using Strauss and Corbin's method and Nvivo software. The reporting follows Consolidated Criteria for Reporting Qualitative Research guidelines.

**Findings:**

The HA role performance theory emerged from data with role enquiry, role dimension, role context, role influence, role reforms and role performance as building blocks. Data analysis showed that the main concerns of the nurses during their daily practice were mediating, speaking up, and negotiating. Among others, the intervening conditions were clientele influence and interpersonal barriers, whereas the outcome was a balance between role reforms and role performance.

**Conclusion:**

Although some nurses proactively initiated biopsychosocial assessment and performed the HA role, most of them relied on clients' requests to perform the role. Stakeholders should prioritise critical thinking during training and intensify mentoring programmes in the clinical areas.

**Relevance for Clinical Practice:**

The present study explains the process by which nurses perform their roles as health advocates in their daily activities as nurses. The findings can be used to teach and guide clinical practice for the HA role in nursing and other health care fields. There was no patient or public contribution.

## 1 INTRODUCTION

The International Council of Nurses (ICN) and the American Nurses Association (ANA) consider advocacy as a vital role of a nurse (ANA, 2010; ICN, 2010). In its position statement on ethics and human rights standards, the ANA (ANA, 2015) declared nurses responsible for advocating for all individuals in the areas of health and healthcare rights. Yet most nurses remain silent when witnessing unsafe practices, with only 10% of nurses speaking up when 50% of nurses described situations that should have resulted in them speaking up (Moss & Maxfield, 2007; Rainer, 2015).

The few who speak up do so without a guide and hence perform the role haphazardly with frustration (Hanks, 2008; Vaartio et al., 2009). This indicates a lack of clarity regarding the description of health advocacy (HA) and the definition of clinicians' scope of responsibility (Hubinette et al., 2017). Consequently, a description of the process of nurses' roles as health advocates is required, particularly in developing countries such as Ghana, a country with a fractured health system (Laari & Duma, 2021), where many rural folks live with an inequitable distribution of health facilities and poor social amenities.

## 2 BACKGROUND

Advocacy, although it originated in legal practice where solicitors spoke on behalf of their clients who could not speak for themselves, is deemed an integral role of the nurse today (Alexis et al., 2022). Advocating for health is an act of pleading in favour of a disadvantaged individual or a community concerning health (Ezeonwu, 2015). It includes educating an individual or group that is disadvantaged to speak out for their rights concerning health and healthcare (Yanicki et al., 2015). Hubinette et al. (2017), in describing differences in approaches to health advocacy (HA) reported a lack of clarity in the description of HA and the definition of clinicians' scope of responsibility. They argued that, regardless of the success of isolated interventions, understanding HA still requires a broader examination of the processes, practices, and values throughout the health system to provide direction for possible HA activities and to establish shared language in communication and collaboration across disciplines.

Over the decades, there have been some general frameworks related to but not specific to HA. As such, a study in Ghana by Adjei et al. (2023) reported there were no specific documented guidelines or framework on advocating for clients, which leaves nurse advocates disappointed and frustrated by failed advocacy attempts.

Carlisle (2000) developed a conceptual framework for health promotion, advocacy, and health inequalities that underscored the concepts of protecting the vulnerable and empowering the disadvantaged. Though essential to an HA role, the study did not address the nurse's HA role performance.

Through synthesising the advocacy literature in nursing, Bu and Jezewski developed a mid‐range theory to clarify and refine the concept of patient advocacy (which is a subset of HA) in 2007, thus establishing a theoretical basis for future studies on patient advocacy in nursing. Their process of synthesising and analysing the advocacy literature identified three core attributes of the concept of patient advocacy, including safeguarding patients' autonomy, acting on behalf of patients, and championing for social justice in the provision of healthcare (Bu & Jezewski, 2007). The core attributes identified by Bu and Jezewski require nurses to be proactive in assessing their clients for information, as suggested by Ceesay (2018). Listening, observing, and questioning skills are important in nursing care assessment to elicit proactive data (Merisier et al., 2018; Phillips et al., 2017). However, most nurses are only seen to react to clients' needs (Rossiter et al., 2017). The high levels of emotional reactivity to issues are debatably linked to a lack of assertiveness within the nursing profession, as reported by Vaupot and Železnik (2018). They reported low levels of assertiveness among nurses towards their clients and other professionals, which they argue prevents some nurses from initiating care. Furthermore, Haley et al. (2017) see active listening as an integral part of nursing and believe that it is significantly associated with empathy, whereas Laging et al. (2018) believe that deficient observation skills during assessment will make subtle issues and changes in the client's condition go unnoticed.

Good listening and observation skills are arguably integral to holistic nursing assessment. However, Fusner et al. (2020), found that even nursing faculty perceive physical assessment skills associated with anatomy and physiological systems as the most critical skills, neglecting the biopsychosocial and spiritual needs of the client. It is argued that bureaucratic barriers (Figueira et al., 2018) and the workload of nurses (Alamri & Almazan, 2018) are responsible for nurses' inability to perform physical assessments in clinical settings to elicit client situations that require advocacy. Though holistic assessment is needed to facilitate the proactive HA role of the nurse, nurses are limited in carrying this out.

Apart from the unassertiveness of some nurses and the lack of biopsychosocial assessment, there are barriers such as the covert nature of HA in the nursing curriculum (van Staden & Duma, 2022), the overlooking and not acknowledging of nurses in some developing countries during health policy development and reviews (Acheampong et al., 2021), and the lack of a framework to guide nurses to perform the HA role (Adjei et al., 2023) are impediments. These issues have culminated in the nurses' silence in performing the HA role, especially in Ghana.

The authors, in structuring the current study for the development of the HA role performance for nurses and to explain the meaning of HA (Burm et al., 2023), considered the arguments by Dang and Dearholt (2017) and Markey et al. (2018). These studies argue for the inclusion of culture and environment as having a important role in shaping nursing practice and should be considered in theory development, and they assert that nurses must remain culturally sensitive in their daily nursing practice. As such, the authors developed a culturally sensitive and contextual HA theory that explains the HA role to empower nurses to understand and embrace the HA role and to answer the question of how nurses understand, accept, and practice their HA role in their daily practice in Ghana.

## 3 METHODS

### 3.1 Design

This is a grounded theory based on Strauss and Corbin (1990). Grounded theory was first advanced by Glaser and Strauss (1967) and later explained by Strauss and Corbin (1990) and Corbin and Strauss (2014). Grounded theory is a systematic methodology used in theory construction that involves inductive, comparative, interactive, and iterative data analysis techniques (Charmaz, 2011). The goal of grounded theory, which is most used in social sciences and qualitative studies, is to understand reality from the perspective of the meaning people place on specific contexts or objects to generate knowledge, improve understanding, and provide an essential guide for action (Morse, 2001). The grounded theory method was chosen since it provided the blueprint for this qualitative study and assisted with inductive theory development by constructing the HA role performance theory from the real‐world environment of the nurses (Strauss & Corbin, 1990). To report our findings, we followed the Consolidated Criteria for Reporting Qualitative Research (Tong et al., 2007).

### 3.2 Setting

This study took place in three public hospitals in three different regions of Ghana. These hospitals were selected purposefully in the northern, middle, and coastal zones among the 16 regions of Ghana. These regional hospitals were selected because they have similar infrastructure and human resources, thus ensuring similarity in the selected hospitals as the research setting yet a diverse cultural setting. The range and scope of nurses' duties in these zones vary depending on the location and culture of the people where each of these hospitals is located. As such, the selection facilitated constant comparison during data analysis, which is a key feature of grounded theory. These hospitals are engaged in training nurses and other health professionals and receive referrals from district hospitals for specialised care. Nurses and midwives working in these facilities are recruited from both the health training institutions (HTI) with 3‐year diploma certificates and the universities with 4‐year bachelor's degrees in nursing. Data were collected between August 2019 and February 2020.

### 3.3 Participants

The inclusion criteria were registered nurses and registered midwives who have worked for more than 5 years in the respective hospitals, irrespective of type of unit. Nurses and midwives who have observed situations requiring HA or who have performed HA during their practice were included. For interviews, a formal meeting for recruitment was organised with assistance from the nurse managers, and full disclosure of the study information was given to the potential participants verbally and in the information sheet. Potential participants asked questions for clarification purposes, and answers were provided by the first author. Participants were asked for voluntary participation, and those who agreed were recruited. In all, 24 nurses and midwives participated, comprising 15 for open sampling and nine for theoretical sampling. The researchers compensated the participants with transportation fares and a snack for those who were off duty but came because of the meeting.

### 3.4 Sampling and data collection

Open and theoretical sampling methods were used to recruit participants for face‐to‐face, in‐depth interviews. These sampling methods are core to grounded theory sampling (Corbin & Strauss, 2014). Open sampling was used based on inclusion criteria to recruit the first 15 participants, consisting of nurse managers and clinical unit heads from each of the three participating regional hospitals. After the initial sample of 15 nurses was taken using open sampling, transcription, and analysis of the data from the initial sample, a theoretical sampling process was initiated. Theoretical sampling is data gathering that is driven by concepts that emerge from an evolving theory and is based on the concept of making comparisons (Corbin & Strauss, 2014). Theoretical sampling was driven by constant comparison of initial data with the purpose of establishing situations and events that maximised opportunities. Theoretical sampling helped in the discovery of variations among concepts and densified categories in terms of their properties and dimensions (Corbin & Strauss, 2008). To achieve this theoretical sampling, an additional three nurses were identified from each of the three participating hospitals, making a total of twenty‐four (24) participants.

The location for the interview chosen by the participants was a nurses' office with the researcher alone. The first author, who was a PhD candidate then and a trained qualitative research male nurse, conducted the interviews using an interview guide developed by the researchers based on the research objectives. These interviews all started with an open‐ended question: “Can you tell me what experiences you have had where health advocacy was used or required to be used?” After the first interview, analysis started, which generated additional questions in further interviews; for instance, “other nurses mentioned victimization as a barrier to advocating; what is your opinion about that?” Each interview lasted between 50 and 70 min and was audio recorded with the participants' permission. During the interview, field notes were taken, which made it easier to remember what was said, where it was, and how nurses acted when they were not talking. The interviews went on until data saturation (Braun & Clarke, 2021).

### 3.5 Data analysis

The recorded interviews were transcribed verbatim, read through, and uploaded into NVivo software to facilitate the data analysis, quick retrieval, and safe storage. Using a grounded theory by Strauss and Corbin (1990), open coding, axial coding, and selective coding, two independent coders (the first author and a qualitative expert) performed a step‐by‐step analysis of the data, and a meeting was arranged with the intercoder to discuss and work on the intercoder reliability score in the presence of the second author. The coding stages between the open and axial coding were performed concurrently, using a recursive line‐by‐line analysis. Open coding was used as a process to discover and identify concepts and their properties from data involving in vivo coding, labelling, and categorisation (Corbin & Strauss, 2014). Open coding was done by recording important concepts and keywords in the research question corresponding to each of the facts raised by the participants using NVivo node options. These were then grouped into individual tables to provide a general picture of the contents of each interview, keeping in mind the research question and objectives. The groups created a preliminary open‐coding framework, which consisted of word descriptors gathered inductively from the data. Similar conceptual abstracts emerging from each interview were organised and grouped following the various codes to make up categories that described nurses' views on HA.

During the analysis, certain questions were asked continuously: What is this study about? What is happening in the data? What is the main idea behind the nurses' practice? Using constant comparison, modifying, and renaming, and sorting memos written, the core category emerged, ‘Role performance’. The arrangement of the core category and other categories represented the initial building blocks of HA theory. For selective coding, data were coded in relation to the core category. A theoretical sampling was carried out to densify the categories. A constant search was done to ascertain a relationship that existed among the concepts from the data, and these concepts were reviewed, compared, validated, and refined. The relationships among the concepts defining nurses' views of HA at different levels of interactional and structural contexts, action strategies, and consequences were then examined. Selective coding was carried out with theoretical coding; memos were sorted, memos on memos were written to increase levels of abstraction and to clarify, integrate, refine, and describe the concepts, using the discriminate sampling process. This discriminate sampling process maximised the opportunity for comparative analysis to integrate the categories along the dimensional level to form a theoretical scheme, validate the statements of the relationship among concepts and fill in any categories in need of further refinement (Corbin & Strauss, 2008) until data saturation.

The first author analysed the data and discussed the codes with the last author, who is experienced in the method of grounded theory and later performed the audit trail. The number of initial codes extracted from the 24 participants was 829, which fell to 385 after repeated codes emerged. The data reduction went on with constant comparison and interaction with the data. Initially, 66 subcategories were extracted and, through continuous refining, reduced to 56. As the data analysis process continued, labels related to the same concepts were put together to form conceptual categories on higher abstraction levels. At the end of the process of concept generation, 55 sub‐subcategories, 16 subcategories, and six categories emerged from the data, as shown in Table 1.

**TABLE 1:** Categorises and subcategories.

Categories	Subcategories
Role enquiry	Listening
	Observing
	Questioning
Role dimension	Proactive advocacy practice
	Reactive advocacy practice
Role context	Institutional advocacy context
	Non‐institutional advocacy context
Role influence	Barriers to advocacy
	Facilitators to advocacy
Role reforms	Training reforms
	Practice reforms
	Practitioner reforms
	Policy reforms
Role performance	Mediating for clients
	Speaking out for clients
	Negotiating for clients

### 3.6 Ethics statement

Ethical approvals were obtained from the Humanities and Social Sciences Research (HSSREC) of the University of KwaZulu–Natal with approval number HSS/0289/018D and the Ghana Health Services Ethics Review Committee with reference number GHS–ERC 007/. Participation was voluntary. Anonymity and confidentiality were adhered to during and after data collection. Before the participants signed the informed consent form, they were given information sheets about the study and told what the goals of the research were.

### 3.7 Trustworthiness

Credibility, confirmability, dependability, and transferability, as described by Lincoln and Guba (1986), were ensured. The credibility of the findings of this study was ensured through member checking by returning to 12 of the participants to read the interview transcripts for scheme validation, whereas for confirmability, transcripts and codes were presented to an intercoder who is an expert in qualitative research for input, and the second author performed an audit trail. Peer debriefing was carried out for confirmability, whereas for dependability, data analysis procedures and software for data analysis procedures are declared. Actual applications of each of the procedures in the study are provided to demonstrate the accuracy of the implementation of the principles of grounded theory. The participants' information, the research context, and the study area are described to allow transferability judgements.

## 4 FINDINGS

The theory presented in this study was based on the findings obtained from the open, axial, and selective coding processes. The means for age and years of clinical experience of the participants were 37 and 11, respectively. These participants included thirteen registered nurses and eleven registered midwives. There were eight participants with diploma certificates: eight with bachelor's degrees, seven with master's degrees, and one with a PhD in nursing. All participants reported having performed the HA role or having observed another nurse performing the role.

The aim of this study was to explore and describe the understanding, acceptance, and practice of the HA role among Ghanaian nurses in their daily practice. In their daily practice, nurses were most concerned with their role performance, which included mediating, speaking up, and negotiating. They described the activities of HA as “role performance,” which was used frequently in their daily practice. Their concerns arose in a role context involving either the institutional advocacy environment or a non‐institutional advocacy environment. In an institutional advocacy environment, situations in which clients were unjustly delayed treatment or nurses noticed medical omissions or misapplications prompted nurses to speak up. Advocating in a non‐institutional advocacy environment, they were concerned about social injustice in communities where the disadvantaged and the less privileged were ignored.

The nurses used various means, such as mediating, speaking up, and negotiating, as a way of advocating, depending on the context and the situation. Some of these situations were not readily apparent, and nurses had to perform biopsychosocial assessment by listening, observing, and questioning to identify issues requiring their intervention, and, as such, they proactively intervened. However, some nurses were not proactive enough to perform a biopsychosocial assessment to identify unmet needs and, as such, were approached by patients or clients who requested assistance regarding their unmet needs. These nurses reacted to the request to intervene on behalf of the patient. Most of the nurses, after identifying the “role context,” such as the situation needing them to intervene and location where this situation occurred, attempted to perform the role but met barriers. Some of these barriers were identified as intrapersonal barriers involving the nurses' innate traits; interpersonal barriers involving relationships with other people; and structural barriers, which had to do with institutional bureaucracy and fear of institutional victimisation. Nurses who met these barriers were unable to perform the role and requested that training, practice, practitioner, and or policy reforms be ensured to provide them with the ability to perform the role.

**FIGURE 1:**
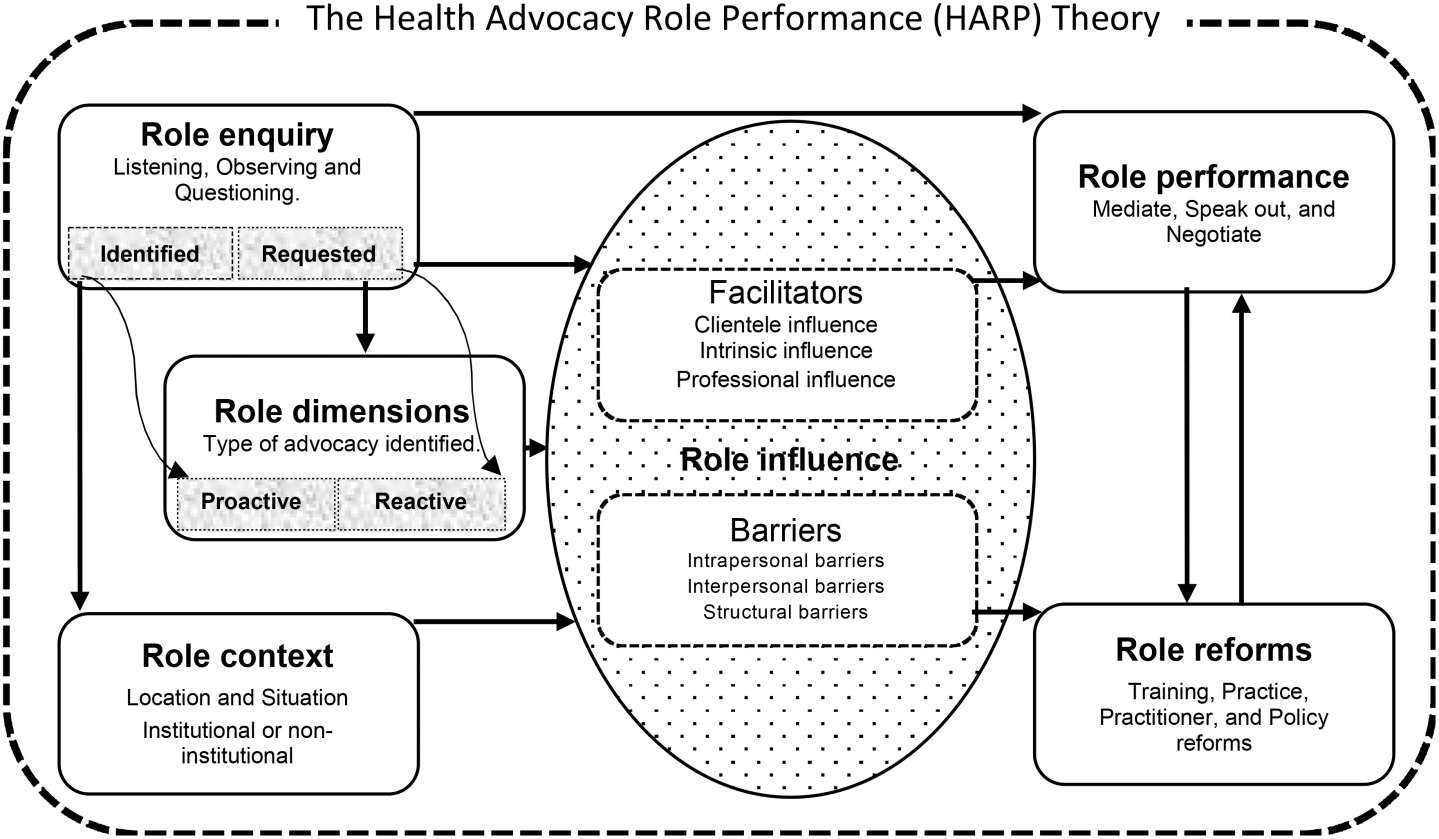
The Health Advocacy Role Performance (HARP) Theory.

However, nurses who, in an attempt to perform the role, where there was professional support from their colleagues, had an intrinsic influence such as self‐motivation and a clientele influence where clients were cooperative with and listened to the nurses, proceeded to perform the role by mediating for, speaking up for, and negotiating for their patients or clients based on the location and the situation of the unmet needs. In the present study, based on the participants' concerns about performing the role and the results of the categorisation process, we selected role performance as the core category that links to all the concept categories from the participants' data. The theory that emerged from the analysis is shown in Figure 1. The details of this process are presented in the following sections.

### 4.1 Main concern: role performance

Role performance emerged as a committed step with mediating, speaking out, and negotiating as dimensions.

#### 4.1.1 Mediating for clients

This is the ability of the nurse to intervene in a disagreement to bring about agreement and reconciliation when the client is involved. Participants mentioned that this involves facilitating communication between individuals and organisations.

When a client comes in, and there are issues with them and other professionals, I mediate because I am supposed to…sometimes from the laboratory or the pharmacy or even from the medical officer. The clients come to us to complain to us, and we mediate (PN21, 31‐year‐old male).

#### 4.1.2 Speaking out

The nurse becomes a voice for the client. Participants noted that they facilitate clients' speaking for themselves and, in some circumstances, they represent the client and speak for them. They speak for the voiceless or do the accompanying speaking, where nurses speak with the voiceless and empower them. It was also pointed out that speaking out for the voiceless in society, for the vulnerable in the community, and for those who have no voice or have their voices not heard is important as a nurse.

I spoke for the boy because his parents were ignorant of the other treatment options available. As an advocate for health, I made them aware of the other options to choose from (PN13, 43‐year‐old female).

#### 4.1.3 Negotiating for clients

Participants mentioned some situations that made them act on behalf of their clients by reaching a compromise while avoiding arguments. Most of the participants reported that negotiating is something they do daily, which involves bargaining with those in power, pleading on behalf of the client, and standing in for the vulnerable.

There was one patient here; after discharge, I had to negotiate…pleaded on his behalf before the administration allowed him to go home (PN23, 51‐year‐old male).

### 4.2 Data analysis for context

Context is a combination of all the circumstances that constitute a situation and includes one's reasons for one's interactions (Corbin & Strauss, 2014). In this present study, the contextual conditions were role context and role influence emerged as the intervening conditions with barriers and facilitators as its dimensions.

#### 4.2.1 Role context

The setting where HA role performance took place and the situations that propelled nurses to stand up or speak out for their clients emerged as role context. The role context emerged, with institutional advocacy context and non‐institutional advocacy context as subcategories.

##### The institutional advocacy context

This was mentioned as a situation where nurses were compelled to speak out for their clients in an institution. The nurses interviewed said they did this when they noticed unfair treatment of clients, including medical omissions and misapplications, unjustly delayed client treatment, and when clients were denied their rights.

Sometimes we go to the authorities because some of the things might involve policies or rules that are hard to break and can lead to unfair treatment of our clients, for instance, deposit before treatment (PN19, 41‐year‐old female).

##### Non‐institutional advocacy context

This was given as examples of times when nurses needed to speak up for a person or group in the nurses everyday lives, not just in a hospital. The nurses did this when they noticed social injustice, such as the unjust treatment of individuals in society. Participants also spoke out to protect the vulnerable and to empower the disadvantaged and less privileged. A participant recounted the non‐institutional advocacy context of a mental health client:

This man (a psychiatric patient) was tied to a tree, and all activities of his daily living were carried out under this tree… We felt it was inhumane. We went to the pastor to advocate for him, and he (the pastor) released the client to be sent for treatment after a long argument concerning the patient's human rights (PN20, 33‐year‐old male).

#### 4.2.2 Role influence

Role influence emerged as the intervening condition that aided or thwarted the nurses' ability to perform their roles as health advocates. Under the role influence, two dimensions emerged: positive intervening conditions called facilitators and negative intervening conditions called barriers.

##### Negative conditions “barriers”

These are the conditions that prevented some nurses from fulfilling the role of HA. Among these were three barriers, including: (1) intrapersonal barriers related to the nurses' innate traits that prevented them from performing the HA role; (2) interpersonal barriers resulting from poor relationships between the client and the professionals, such as poor clientele traits and persecution from colleges; and (3) structural barriers such as organisation and management issues, including red tape, poor educational preparation, professional alienation, and victimisation from the authorities (Laari & Duma, 2023b). A participant reported that:

Clients themselves are part of it. I have stood up for clients several times, but at the end of the day, some of them behaved in a way that made it difficult for me to advocate for them in the future (PN16, 31‐year‐old female).

##### Positive conditions “facilitators”

These conditions emerged as ones that aided nurses in their HA roles. These facilitators included: (1) clientele influence, which relates to the readiness and openness of the client to receive assistance from the nurse; (2) the intrinsic influence of the nurse, where the nurse is self‐motivated and driven by empathy and compassion to speak out for the client; (3) professional influence, where the nurse sees advocacy as a professional obligation that links with years of experience and the educational background of the nurse; and (4) cultural influence relating to the nurse's religious background, where they perform advocacy as a religious obligation (Laari & Duma, 2021).

For me, I see it as a religious obligation; yes, to speak up for someone and help them get what they couldn't get on their own is a privilege for me as a religious person (PN20, 33‐year‐old male).

### 4.3 Data analysis for process

The action/interaction strategies with regard to role performance were role enquiry and role dimensions.

#### 4.3.1 Role enquiry

Role inquiry is when a nurse gathers information about a client or does a biopsychosocial assessment of a client to figure out if the client needs the nurse to play a HA role. It is an important step for the nurse to take in order to find out what the client's unmet needs are.

So, I started talking to her. I asked her if she had any information about her health, concerning her status, and she gave us information that was very necessary for her care. Enquiry from the client has always been one thing I consider very important prior to client care (PN11, 41‐year‐old male).

Observing, listening, and questioning emerged under role enquiry.

##### Observing the client

This is the nurse's ability to watch the client during care to help ascertain issues that require advocacy. The nurses' observational skills allowed them to assess not only the clients' unmet needs but also the immediate and remote environments of the clients.

We are always observant of the patient's reactions to issues, and mostly it gives us clues and information for some action to be taken (PN24, 33‐year‐old male).

##### Listening to the client

This involves the accuracy of the nurse in receiving verbal and nonverbal information from the client during communication. Listening keenly assisted the nurses in identifying their clients' situational needs and advocating for them.

I try to have a keen interest in what they tell me, so I listen keenly all the time I communicate with my clients (PN13, 43‐year‐old female).

##### Questioning the client

The ability of the nurse to make relevant inquiries that would reveal the issues of the client that would require the nurse to speak up for. This, according to the participants, was done by interviewing clients for information and conferring with other professionals to get the information necessary for clients' care. The nurses reported that questioning serves many functions during client care in healthcare facilities.

I do ask my patients questions, sometimes in the form of interviews or during personal interaction. By asking them some relevant questions, I get to know and understand them better, and understanding them helps me in my care for them (PN01, 33‐year‐old male).

#### 4.3.2 Role dimensions

These are dimensions of HA that nurses perform. Proactive HA and reactive HA emerged as subdimensions. A participant noted that:

I believe in the rights of customers, whether they ask for help or not… we have demanded an unqualified apology for a lot of our clients here when they were abused by other professionals without the client's knowledge… but in some cases the clients requested for us to intervene (PN04, 37‐year‐old female).

##### Proactive health advocacy

A situation during HA role performance where the nurse monitors, controls, or performs an action based on his or her independent biopsychosocial nursing assessment of the client without a demand from the client. The nurses saw this as a preventive process in the client's care where they championed clients' rights when patients were abused and not treated well. They initiate and do not wait until clients complain to them because some clients will never request help.

Sometimes issues pushed us to the point where we spoke language that sounded like threats to management before things were done. If the most basic things are not there and all you are telling me is to manage, manage with what?… I value the lives under my care, so I usually don't wait for clients to complain or request (PN13, 43‐year‐old female).

##### Reactive health advocacy

This is a response to a request from the client for the nurse to perform the HA role. The nurses spoke out to facilitate access to care or to provide the requested service in the form of leading and teaching the client. The participants saw this as a curative process in the client's care.

We felt she was not treated right concerning her bills, so when she came and laid the complaints, we responded and followed up with the right offices to make sure that all her needs regarding her healthcare were met (PN03, 36‐year‐old female).

### 4.4 Consequence

The findings of this study showed that the consequence of the nurses action‐interaction in relation to role performance was role reforms. This category consisted of training, practice, practitioner, and policy reforms.

#### 4.4.1 Role reforms

These are the changes and strategies needed to reform in the right direction for better accomplishment. The people who took part said that role reforms were ways to recheck and fix things that needed to be changed. Training, practice, practitioner, and policy reforms were the four areas that required reform.

##### Training reforms

Participants mentioned that incorporating necessary changes in the professional education process will enable students to be exposed to HA while they are in school. The nurses believe that changes that are important in training reform are to incorporate HA into the training curriculum, have curriculum innovations, and encourage lifelong learning concepts during the training (Laari & Duma, 2023a).

If they (authorities) could add health advocacy to the curriculum at the nursing training school… it would help a lot to get the trained nurse to acquire the necessary knowledge and the attitude to work on the health advocacy role for clients (PN23, 51‐year‐old male).

##### Practice reforms

These are the changes that are needed in the professional culture to facilitate the performance of HA. As reported by the participants, continuous professional education, interprofessional collaboration, and empowering nurses with advocacy skills to change negative professional socialisation are needed within the practice, as mentioned by the participants (Laari & Duma, 2023a).

There should be a clear pathway so that we will know the right place to go when something is happening. But for now, we don't know whom to go to for what, and it makes the performance of the health advocacy role cumbersome (PN13, 43‐year‐old female).

##### Practitioner reforms

These are the changes an individual nurse or group of nurses requires to facilitate and promote the performance of the HA role. Participants reported the need for attitudinal change among professionals, enhancement of professional solidarity, empowerment of professional assertiveness, and professional approachability (Laari & Duma, 2023a). A participant reported how unity and professional solidarity can reform the practitioner.

Our colleagues with experience in health advocacy who know what to do should mentor the young nurses and coach them to grow up. Uniting will make our role great. (PN01, 33‐year‐old male).

##### Policy reforms

Participants mentioned the regulatory bodies, such as the Nursing and Midwifery Council of Ghana, the Ministry of Health, the Health Training Institutions, and the implementing bodies, including the Ghana Health Services and the Christian Health Association of Ghana, as key stakeholders. These bodies were thought of by participants as bodies that should provide reformed policies that are necessary for the policy reforms and provide a supervisory role for the reforms to be successful (Laari & Duma, 2023a).

Nurses who work as health advocates need clear policies and rules to guide and protect them. Those in charge up there should restructure (reform) our policies. (PN01, 33‐year‐old male).

### 4.5 Assumptions of the theory

The HARP theory assumes that the HA role of the nurse will facilitate healthcare equity in underdeveloped and developing countries. Nurses are the frontline staff in healthcare, and if they are equipped with the necessary knowledge and skills to protect the vulnerable and empower the disadvantaged and less privileged, health for all would be a reality. It is assumed that providing proper knowledge to nurses today, either through in‐service or formal education in HA, is an investment that would bring huge dividends in the future for society.

## 5 DISCUSSION

The aim of this study was to identify and explain how nurses perform their HA role in underserved populations. The HARP theory describes how nurses perform their HA role in underserved populations. Varied categories of nurses were interviewed across Ghana in three strategic locations. Nurses of various ages, varied experiences, and vast ranges of practical experiences were sampled, which gives this theory densified data (Dearholt & Dang, 2012; Markey et al., 2018).

This HARP theory is different from role theory by Turner (2001), and social theory and social structure by Merton and Merton (1968). The HARP theory uncovers and describes nurses' practices in underserved populations. The current findings reveal most nurses see HA as a professional obligation and perform this role with keen interest. Though some professional nurses only reacted to their clients' needs and were not proactive enough to assess and initiate the HA role for their clients, their responses confirm the assertions by ANA and ICN that nurses top the list of professionals who speak for their clients (ANA, 2010; ICN, 2010).

Few people who did assessments did so with little to do with their clients' HA needs. Instead, they looked at their clients' anatomy and physiology to find problems. This resulted in some participants not performing holistic nursing care for their clients, as previously argued by Fusner et al. (2020), that most faculty perceiving assessment skills associated with the human anatomical systems as the most critical skills taught in nursing assessment courses. Again, a previous study by Alamri and Almazan (2018) reported that nursing students did not practice assessment in the clinical setting, although they were oriented and educated about assessment in the nursing curriculum. This inability to perform assessments appears to be linked with workload and red tape issues, as identified in this current study. Though assessment is a key skill in nursing practice, the value of nursing assessment is poorly recognised, and there is a lack of clarity regarding the importance of nursing assessments (Ceesay, 2018; Laging et al., 2018).

Biopsychosocial assessment should be encouraged from the undergraduate level on to instil a culture of role inquiry in future nurses, as it will aid in the development of skills to provide holistic and person‐centred care. It should be encouraged because partial and incorrect performance of patient assessment promotes less critical thinking and could lead to poor clinical decisions (Alamri & Almazan, 2018).

The current findings identified role enquiry to supports previous literature where listening, observing, and questioning were seen as important in nursing care assessment, as stated by Merisier et al. (2018) and Phillips et al. (2017). Haley et al. (2017) also, reported on the significance of active listening as an integral part of nursing when they identified that active listening was significantly associated with empathy. Similarly, the current study reveals that listening, observing, and questioning are important in nursing role performance. Without keen listening and attentive observation, subtle issues and changes in the client's condition can go unnoticed.

As averred by Rossiter et al. (2017), nurses play an age‐old role in reacting to clients' unmet needs or responding to clients' needs. However, because proactive HA appears to be lacking in the current findings, it is past time for nurses to perceive and act as proactive professionals. Proactive advocacy allows the nurse to initiate and perform the HA role without a request from the client, which facilitates the preventive roles of nurses. The current findings show that more nurses perform reactive advocacy than proactive advocacy, suggesting one of two things. Either these nurses had insufficient knowledge to advocate or they were unassertive to initiate the HA role performance process. A question that requires an answer in a further study is: What promotes proactive activity among nurses during professional duties? The seeming lack of assertiveness within the nursing profession is reported by Vaupot and Železnik (2018), who believe low levels of assertiveness among nurses towards their clients and other professionals are evident. Further education of the nurse might be necessary to increase and improve knowledge to promote assertiveness as the current findings, although not conclusive, reveal that nurses with further education and important years of experience in nursing practice are assertive and inclined to advocate for their clients.

Nurses who identified or got requests from their clients performed one or more of the following: mediating, speaking out, or negotiating. Speaking for clients has always been a role of the nurse. As such, the current finding confirms the role of nurses as advocates who speak out when there is a medical omission or when client rights are ignored during treatment. This is consistent with Bu and Jezewski (2007) findings that nurses safeguard patients' autonomy, act on behalf of patients, and champion social justice in the provision of health care. Some conditions, such as clientele influence, professional influence, cultural influence, and intrinsic influence, promote the performance of the HA role (Laari & Duma, 2021). The presence of these influencers made it easier for the nurse to perform their HA role. Divergently, there were barriers, such as the covert nature of advocacy in the curriculum, as previously averred by Alamri and Almazan (2018) and Douglas et al. (2018), that prevented nurses from advocating.

The need for key reformations was uncovered, and policymakers are encouraged to use these reformations to enhance clear implementation strategies in HA (Laari & Duma, 2023a). This included training and practice reforms. It, however, appeared as a challenge as nurses are not involved in most policy development that affects them, as reported by Acheampong et al. (2021) in Ghana, where nurses are usually overlooked and not acknowledged during health policy development and reviews. The best way to move forward is to ask nursing experts to help make and review policies. This will give experts the knowledge they need to make changes.

### 5.1 Relevance to clinical practice

The current findings reveal most nurses are inclined to react to situations rather than proactively initiating a biopsychosocial assessment. Nurse educators should prioritise critical thinking during nurses' training to incorporate assertiveness and the ability to initiate within their job description without requests from clients. The idea of biopsychosocial nursing assessment should be emphasised as a way to encourage nurses to keep learning to improve their skills through in‐service training. Mentoring and coaching should be encouraged in the clinical areas.

### 5.2 Limitation and recommendations for further research

This current study described how nurses performed their HA role in underserved populations. Due to contextual and cultural differences that might affect the behaviours and actions of nurses in developed countries, further research to modify this theory for other cultural and economic contexts is necessary. Although, we recruited from three zones in the country that facilitated constant comparison, nurses in private health facilities who met the inclusion criteria were not recruited, views from these professionals would have enhance the findings. Future studies should consider comparing HA activities in both private and public hospitals, and a clear relationship between the nurse's education and the concept of role dimensions should be explored. The inclusion criteria considered nurses and midwives with more than 5 years work experience; this was purported to recruit participants with rich HA role experience. Some participants in these excluded categories could have given an insight view relevant to this study.

## 6 CONCLUSION

The HARP theory suggests that the role inquiry, which dealt with biopsychosocial assessment, is the driving force behind HA role performance. The theory systematically presented a descriptive pathway of how nurses and midwives perform the HA role, beginning with role inquiry, proceeding through role dimensions, role context, and role influence, and terminating at either role performance or role reforms. As some nurses proactively initiated their professional obligation to perform the role, others were thwarted from carrying out their roles as advocates due to barriers.

## AUTHOR CONTRIBUTION

Both LL and SED conceptualised the idea, wrote the proposal, and designed the instruments for data collection. LL collected the data and analysed whiles SED reviewed and made corrections. LL and SED read and approved the manuscript. Both authors contributed to this paper.

## FUNDING INFORMATION

The authors did not receive any funding for this manuscript.

## CONFLICT OF INTEREST STATEMENT

Both authors declare no conflict of interest.

## Data Availability

The data that support the findings of this study are available from the corresponding author upon reasonable request.
